# Sonographically derived tissue characteristics and functional performance adaptation across a competitive season in division I female volleyball athletes

**DOI:** 10.3389/fspor.2026.1957488

**Published:** 2026-10-27

**Authors:** Nathan J. Savage, Troy Purdom, Chris Wendt

**Affiliations:** 1 Department of Physical Therapy, High Point University, High Point, NC, United States; 2 Department of Kinesiology, North Carolina Agricultural and Technical State University, Greensboro, NC, United States; 3 Department of Physical Therapy, Winston-Salem State University, Winston-Salem, NC, United States

**Keywords:** athlete monitoring, jump performance, sonography, tendinopathy, ultrasound imaging

## Abstract

**Introduction:**

Diagnostic neuromusculoskeletal ultrasound imaging (USI) is increasingly used in athletic populations to characterize tendon and enthesis structure and to monitor seasonal change; however, the functional significance of sonographic tissue characteristics and their relationship to performance remain unclear. This study examined the association between sonographic tissue characteristics and performance measures in Division I female volleyball athletes.

**Methods:**

Athletes from a single team underwent pre-season (*N* = 16) and post-season (*N* = 15) USI examination of bilateral shoulder, anterior knee, posterior ankle, and plantar foot regions, along with standardized performance testing. Continuous sonographic measures included supraspinatus tendon and plantar fascia thickness and patellar and Achilles tendon cross-sectional area; categorical pathology indicators included the presence or absence of tendinopathy and enthesopathy. Performance outcomes included single-leg hop distance and single-leg vertical jump height, double-limb vertical jump, drop jump, broad jump and double broad jump performance, jump-landing movement quality, and closed kinetic chain upper extremity stability. Linear mixed-effects models were used to evaluate associations with performance level and seasonal change.

**Results:**

Performance measures demonstrated modest, task-dependent seasonal changes. Continuous and categorical sonographic tissue measures were not meaningfully associated with performance level or seasonal performance change. Contextual monitoring variables were not associated with double-limb performance and did not modify seasonal performance adaptation. Changes in corresponding right and left tendon measures were strongly correlated, particularly for Achilles tendon cross-sectional area; however, these structural characteristics showed weak correspondence with functional performance adaptations.

**Discussion:**

In Division I female volleyball athletes, seasonal performance adaptation was modest, while sonographic tissue characteristics showed limited association with performance. Tissue adaptation demonstrated weak correspondence with functional performance change, suggesting that structural and performance adaptations may follow partially independent trajectories across a competitive season. These findings support interpreting USI findings within a broader clinical and performance context.

## Introduction

Division I collegiate volleyball places substantial and repetitive mechanical demands on the lower and upper extremities, driven by frequent jumping, landing, and overhead hitting activities (1). These demands are associated with a high prevalence of tendon and enthesis-related adaptations, particularly involving the patellar and Achilles tendons (2, 3), structures that are associated with knee and ankle injuries and may result in substantial time loss and career impact in collegiate-level athletes (4). In the evaluation of orthopedic-related complaints, diagnostic ultrasound imaging (USI) is routinely employed to provide high resolution visualization of neuromusculoskeletal tissues, including the commonly injured patellar and Achilles tendons. Consequently, USI is increasingly used in athletic populations to describe tissue structure, monitor seasonal change, and inform clinical decision-making (5, 6). However, the functional significance of sonographic tissue characteristics—particularly their relationship to athletic performance and its seasonal adaptation—remains unclear (7, 8).

Prior research in volleyball and other jumping sports has demonstrated a high prevalence of qualitative sonographic tendon abnormalities in asymptomatic athletes, including hypoechoic thickening, loss of normal fibrillar architecture, and intrasubstance defects, both before and after a competitive season (2, 9, 10). Yet evidence linking these findings to performance capacity, performance change, or injury risk is inconsistent (3, 11, 12). Large cohort and meta-analytic studies evaluating qualitative grayscale tendon abnormalities suggest that these findings may increase future pain risk; however, definitions of abnormality vary across studies, their positive predictive value is low, and their functional relevance at the time of detection remains uncertain (9, 13–15). Much of the existing literature has focused on cross-sectional descriptions of tendon morphology or pathology prevalence, often without concurrent assessment of functional measures such as jump height, hop distance, landing movement quality, or closed kinetic chain upper-extremity performance, or has examined structural adaptation without directly evaluating contemporaneous performance (10, 16, 17). Consequently, it remains uncertain whether sonographic measures reflect adaptive, maladaptive, or clinically neutral tissue responses to seasonal loading, or whether they meaningfully correspond to functional performance measures (5, 8).

Athlete monitoring variables—including movement-quality assessments and self-reported measures of stress, recovery, and energy availability—are frequently used alongside performance testing to contextualize training response and injury risk. Tools such as the Landing Error Scoring System (LESS), the Recovery–Stress Questionnaire for Athletes (RESTQ-36-Sport), and the Low Energy Availability in Females Questionnaire (LEAF-Q) have demonstrated utility for screening and monitoring purposes (18–23). However, their ability to explain or modify relationships between tissue structure and performance across a competitive season has not been well established, particularly when evaluated concurrently with sonographic measures rather than as independent screening tools.

Additionally, limited work has examined whether seasonal changes in sonographic tissue characteristics covary with changes in performance, or whether tissue adaptations demonstrate bilateral or regional coupling independent of functional measures (24, 25). Notably, studies directly examining connective tissue morphology in relation to jump-based performance have reported weak or absent associations, despite clear sport-specific and region-specific structural adaptations (24, 25). Understanding these relationships is critical for interpreting USI findings in high level athletes and for determining whether structural imaging markers provide information beyond that obtained from performance testing and athlete-reported monitoring tools.

This cohort has been previously described with respect to seasonal sonographic findings and body composition (10); the present study extends that work by evaluating whether sonographic tissue characteristics are associated with limb-specific and bilateral performance measures across a season, addressing a gap not examined in prior prevalence-focused or risk-focused USI studies. Therefore, the purpose of this prospective observational study was to examine the associations between sonographic tissue characteristics and performance measures across a single NCAA Division I women's volleyball season. Given the unique physiological and sport-specific demands experienced by female volleyball athletes, we specifically evaluated whether continuous measures of tendon thickness and cross-sectional area and categorical indicators of tendinopathy and enthesopathy were associated with single-leg hop and vertical jump performance, double-limb jump performance, jump-landing movement quality, and closed kinetic chain upper extremity performance. We also evaluated whether the sonographic measures moderate pre-season vs. post-season performance change and whether contextual monitoring variables influenced these relationships. Secondary analyses explored tissue asymmetry and interrelationships among sonographic measures. We hypothesized that associations between sonographic tissue characteristics and performance outcomes would be limited and that seasonal performance adaptation would be driven primarily by inter-individual differences rather than structural imaging markers.

## Materials and methods

This prospective observational repeated measures study was conducted across one NCAA Division I volleyball season. Sonographic tissue characteristics were examined as continuous measures of supraspinatus tendon and plantar fascia thickness and patellar and Achilles tendon cross-sectional area, and as binary indicators of tendinopathy and enthesopathy.

Primary outcomes were limb-specific composite lower extremity performance scores derived from standardized single-leg hop distance and single-leg vertical jump height. Secondary outcomes included the corresponding single-leg component measures; composite and component double-limb vertical jump, drop jump, broad jump and double broad jump performance; jump-landing movement quality assessed with the LESS; upper extremity performance was assessed using the Closed Kinetic Chain Upper Extremity (CKCUE) Stability Test; and athlete-reported RESTQ-36-Sport and LEAF-Q measures. The protocol was approved by Winston-Salem State University (IRB-FY2023-79); all athletes provided written informed consent.

### Participants and procedures

Athletes from a single NCAA Division I female volleyball team at the largest Historically Black University in the United States were recruited. Athletes were included if medically cleared for full participation and excluded for inability to complete testing or time loss injury at either follow-up assessment.

At baseline, athletes completed a study-specific demographic and health history questionnaire that recorded age, dominant hand, race and ethnicity, current musculoskeletal pain and its location, and musculoskeletal surgery during the preceding year. Responses were summarized descriptively and were not combined into a composite health history score. Pre-season assessments were conducted approximately one week before the start of the competitive season and post-season assessments were completed within one week following the final competitive contest. Pre-season and post-season assessments included USI examinations, lower and upper extremity performance testing, and athlete-reported measures using standardized procedures and identical equipment. The two assessments were separated by 12 weeks.

### Sonography and image analysis

Image acquisition, participant positioning, measurement landmarks, and normalization procedures were consistent with our previously published protocol in this cohort, which includes representative figures of participant positioning and image acquisition (10). Sonographic examinations were performed by the principal investigator (Registered in Musculoskeletal® sonography credential; >9 years’ experience performing, teaching, and researching neuromusculoskeletal USI) using a 15–4 MHz linear-array transducer (Sonosite PX; FUJIFILM Sonosite).

USI examinations included bilateral assessment of the supraspinatus tendon and humeral insertion in the shoulder region, the patellar tendon and patellar and tibial insertions in the anterior knee region, the Achilles tendon and calcaneal insertion in the posterior ankle region, and the plantar fascia and calcaneal insertion in the plantar foot region. For the shoulder region, athletes were seated and in a modified Crass position to obtain long-axis and short-axis views of the supraspinatus tendon and insertion. For the anterior knee, athletes were supine with their knees flexed ≈ 30° over a bolster to obtain long-axis and short-axis views of the proximal and distal portions of the patellar tendon and insertion. For the posterior ankle and plantar foot regions, athletes were prone with their distal legs and feet off the table to obtain long-axis and short-axis views of the Achilles tendon and insertion, and long-axis views of the plantar fascia and insertion. Each region was evaluated for tendon abnormalities, including hypoechoic thickening, altered fibrillar architecture, and intrasubstance defects, and for enthesis-related abnormalities, including osteophytes, enthesophytes, and cortical irregularities.

Images were exported and analyzed using ShowCase Premiere (v6.1.3.11; Trillium Technology) with standardized landmarks and planes. Thickness was measured for the supraspinatus tendon (superior facet of the greater tubercle) and the plantar fascia (distal calcaneal curvature); cross-sectional area (CSA) was measured for the patellar (proximal and distal thirds) and Achilles tendons (malleolar intersection). These variables were selected because tendon thickness and CSA are among the most commonly reported sonographic measures of tendon and connective tissue morphology. CSA was used for the patellar and Achilles tendons because their transverse borders could be delineated at standardized anatomical landmarks, whereas thickness was used for the supraspinatus tendon and plantar fascia in accordance with established structure-specific measurement approaches (5, 26, 27). Sonographic measures were normalized to one-third body mass, consistent with the previously published analysis of this cohort and other studies using this referenced normalization approach (10, 24). This scaling procedure was intended to reduce between-athlete anthropometric variability and facilitate comparisons across athletes of differing body sizes.

All USI examinations and measurements were performed by a single experienced examiner using standardized acquisition and analysis procedures. Study-specific intra-rater reliability was not evaluated for the sonographic measurements. However, similar tendon thickness and CSA measurement protocols have demonstrated good to excellent reliability in previous studies (5, 26–28).

### Sonographic tissue pathology

Tissue pathology was coded as present or absent for each body region; severity was not graded. The principal investigator classified each image during image review using predefined qualitative sonographic criteria rather than structure-specific numeric thresholds. Tendinopathy was defined by hypoechoic thickening, loss of normal fibrillar architecture, and/or an intrasubstance defect (2, 5, 9, 11, 13). Enthesopathy was defined by the presence of an osteophyte, enthesophyte, and/or cortical irregularity at the enthesis (5, 10, 15, 29).

### Performance measures

After a standardized dynamic warm-up, athletes completed performance testing including single-leg vertical jumps, single-leg hops, vertical jumps, 30 cm drop jumps, broad jumps and double broad jumps, and the CKCUE Stability Test. For horizontal jumping tasks, performance was quantified as jump distance (cm), whereas vertical jumping tasks were quantified as jump height (cm) derived from flight time. The CKCUE Stability Test quantified the number of alternating hand touches (line 91 cm apart) completed in a standardized push-up position over 15 s (30).

Vertical jumping tasks were simultaneously video recorded in the frontal and sagittal planes using two 60 Hz cameras and later analyzed using Kinovea (v0.9.5), a method previously shown to provide valid and reliable estimates of vertical jump performance. Takeoff was identified as the first frame in which the foot or feet were no longer in contact with the floor, and landing was identified as the first subsequent frame in which floor contact was re-established. When the exact event occurred between two consecutive frames and could not be confidently assigned to a single frame, the event time was estimated as the midpoint between the two frames. Flight time was calculated from the elapsed time between takeoff and landing and used to determine jump height according to the standard projectile motion equation (height = gravity × time^2^/8), simplified to: height (cm) = 122.6 × t^2^, where t represents flight time in seconds (31). For each jumping task, the mean jump height across three maximal trials was used for statistical analysis.

Within-session reliability of all pre-season performance measures was evaluated using repeated trials with intraclass correlation coefficients (ICC_3,k_) using a two-way mixed, absolute agreement, and average measures approach.

### Athlete-reported measures

Athletes completed the RESTQ-36-Sport and LEAF-Q electronically prior to each session; scores were modeled as contextual predictors and potential moderators of performance change (18, 21).

### Statistical analysis

Analyses were conducted in SPSS v30.0.0.0 using linear mixed-effects models (random intercept for athlete; variance components; REML; Satterthwaite df; Type III tests). Primary models tested Time, sonographic predictors, and Time × predictor terms for limb-specific outcomes.

For the primary analyses, single-leg hop distance and single-leg vertical jump height were standardized separately for each limb using the pooled mean and standard deviation calculated across all available pre-season and post-season observations. The resulting z-scores were averaged to form limb-specific composite performance scores, with higher values indicating better overall performance. This approach was selected to place the two performance tests on a common scale and to generate a single summary measure of lower extremity explosive performance. Because standardization was based on the pooled distribution of observations rather than individual baseline values, changes in composite scores should be interpreted as changes in an athlete's relative performance position within the cohort rather than as direct measures of absolute within-athlete performance change. Consequently, an athlete could demonstrate stable raw performance values across the season while exhibiting a small change in composite score if the overall group distribution shifted between assessments. To address this limitation and facilitate interpretation of absolute performance change, the individual component outcomes were also analyzed and reported separately, particularly because this specific composite has not been independently validated. Single-limb analyses were conducted for composite and individual component measures. The upper extremity analysis normalized CKCUE Stability Test scores to athlete height and analyses were conducted for average supraspinatus tendon thickness for both shoulder and individual component thickness measures (22).

Secondary models evaluated composite and component double-limb (vertical jumps, drop jumps, broad jumps and double broad jumps) measures and sonographic asymmetry predictors and contextual predictors (LESS score, RESTQ-36-Sport, LEAF-Q) with Time × predictor terms. Model fit was summarized using model-based ICC (variance partition coefficients) and pseudo-*R*^2^. Mixed-effects models used all available observations; correlations used Spearman rho with pairwise deletion of missing values.

Generative artificial intelligence (Microsoft Copilot) was used to assist with manuscript preparation, including language refinement and organizational clarity. No artificial intelligence tools were used for data collection, image acquisition, image interpretation, statistical analysis, or generation of results. All analyses, interpretations, and conclusions were performed by the authors.

## Results

Complete sonographic and performance data were collected on 16 athletes at pre-season and 15 athletes at post-season due to team departure. An additional athlete reported left knee soreness at post-season testing and did not complete select left limb performance tasks. Descriptive summaries and exploratory paired pre-season vs. post-season comparisons of athlete characteristics, performance measures, sonographic measures, and categorical pathology indicators are presented in Tables 1, 2. Because these analyses included multiple outcomes and the pilot sample was small, the findings should be interpreted as exploratory. Primary mixed-effects models showed strong athlete-level clustering and no evidence that sonographic tissue characteristics (continuous or categorical) modified composite or component limb-specific performance trajectories. Composite outcomes reflect standardized relative performance within the study cohort; therefore, component outcomes are presented alongside composite findings to aid interpretation of absolute performance changes.

**Table 1:** Descriptive summary of pre-season and post-season continuous variables.

Variable	*N*	Pre-Season Mean ± SD	*N*	Post-Season Mean ± SD	*Δ* Mean (Post–Pre)	*P* Value^‡^
Participant Characteristics						
Age (years)	16	19.91 ± 1.22	15	20.19 ± 1.19	+0.28	NA
Height (cm)	16	174.94 ± 8.35	15	175.13 ± 8.90	+0.19	.215
Body mass (kg)	16	73.24 ± 9.41	13	74.86 ± 8.27	+1.62	.567
Resting heart rate (bpm)	16	74.81 ± 12.23	13	77.92 ± 10.17	+3.11	.290
Systolic pressure (mmHg)	16	117.13 ± 9.39	13	119.08 ± 10.81	+1.95	.574
Diastolic pressure (mmHg)	16	75.81 ± 6.48	13	63.85 ± 7.41	−11.96	<.001
Athlete-Reported Measures						
RESTQ-36-Sport score	16	33.44 ± 5.35	15	31.61 ± 4.66	−1.83	.386
LEAF-Q score	16	8.13 ± 3.54	12	6.42 ± 3.15	−1.71	.403
Performance Measures						
Single-leg hop (cm), Right	16	152.32 ± 18.79	15	148.06 ± 22.02	−4.26	.652
Single-leg hop (cm), Left	16	149.49 ± 22.67	14	178.36 ± 25.00	+28.87	<.001
Single-leg vertical jump (cm), Right	16	20.88 ± 3.88	15	19.14 ± 3.44	−1.74	.085
Single-leg vertical jump (cm), Left	16	20.56 ± 4.17	14	18.11 ± 2.75	−2.45	.006
Broad jump (cm)	16	197.56 ± 20.33	15	191.85 ± 16.24	−5.71	.409
Double broad jump (cm)	15	399.49 ± 30.77	15	380.38 ± 33.62	−19.11	.027
Vertical jump (cm)	16	39.97 ± 6.11	15	36.14 ± 4.28	−3.83	.001
Drop jump (cm)	16	38.52 ± 5.64	15	34.72 ± 2.93	−3.80	.004
CKCUE stability (reps)	16	21.13 ± 3.25	15	23.87 ± 3.31	+2.74	<.001
LESS score, Total	16	4.65 ± 2.09	14	5.41 ± 2.45	+0.76	.278
LESS score, Frontal plane	16	3.34 ± 1.40	15	3.51 ± 1.52	+0.17	.799
LESS score, Sagittal plane	16	1.33 ± 1.45	14	1.90 ± 1.34	+0.57	.190
Single-leg hop symmetry (%)	16	94.30 ± 4.49	15	83.96 ± 8.88	−10.34	.003
Single-leg vertical jump symmetry (%)	16	92.49 ± 6.63	15	94.19 ± 5.36	+1.70	.507
Sonographic Measures^†^						
Supraspinatus tendon thickness (mm), Right	16	14.60 ± 1.85	15	14.63 ± 1.91	+0.03	.864
Supraspinatus tendon thickness (mm), Left	16	13.12 ± 2.01	15	13.30 ± 1.97	+0.18	.974
Patellar tendon, proximal CSA (mm^2^), Right	16	20.44 ± 3.35	15	22.29 ± 4.02	+1.85	.055
Patellar tendon, proximal CSA (mm^2^), Left	16	19.63 ± 3.67	15	21.12 ± 3.80	+1.49	.205
Patellar tendon, distal CSA (mm^2^), Right	16	19.97 ± 3.81	15	19.39 ± 3.24	−0.58	.134
Patellar tendon, distal CSA (mm^2^), Left	16	19.17 ± 3.62	15	20.75 ± 3.78	+1.58	**.046**
Achilles tendon CSA (mm^2^), Right	16	12.62 ± 1.65	15	14.23 ± 1.98	+1.61	**<.001**
Achilles tendon CSA (mm^2^), Left	16	12.29 ± 1.53	15	13.28 ± 1.79	+0.99	**.006**
Plantar fascia thickness (mm), Right	16	4.14 ± 1.12	15	4.87 ± 1.01	+0.73	**.004**
Plantar fascia thickness (mm), Left	16	4.41 ± 1.08	15	4.89 ± 0.95	+0.48	**.006**

bpm, beats per minute; mmHg, millimeters of mercury; RESTQ, Recovery-Stress Questionnaire; LEAF-Q, Low Energy Availability in Females Questionnaire; VJ, vertical jump; CKCUE, closed kinetic chain upper extremity; LESS, Landing Error Scoring System; CSA, cross-sectional area.

Bold indicates *P* < .05 (NA indicates statistic could not be calculated).

† Normalized to one-third body mass.

‡ *P* values represent paired pre-season vs. post-season comparisons among athletes with complete data at both assessments.

**Table 2:** Descriptive summary of pre-season and post-season categorical variables.

Variable	Pre-Season N (%)	Post-Season N (%)	*P* Value^†^
Participant Characteristics			
Dominant hand, Right	14 (88)	13 (87)	.945
Ethnicity, Hispanic	1 (6)	1 (7)	.962
Race, Black or African American	9 (56)	8 (53)	.870
Currently experiencing MSK pain, Yes	8 (50)	7 (47)	.853
Pain location, Lower limb(s)	6 (38)	6 (40)	.886
MSK-related surgery in past year, Yes	1 (6)	1 (7)	.962
Location of surgery, Lower limb(s)	0 (0)	0 (0)	NA
Athlete-Reported Measure			
LEAF-Q score, High risk (≥8 points)	8 (50)	3 (25)	.180
Performance Measure			
LESS score, High risk (≥5 points)	7 (44)	10 (71)	.127
Sonographic Evidence of Tendinopathy			
Supraspinatus, Right	6 (38)	8 (53)	.376
Supraspinatus, Left	5 (31)	9 (60)	.108
Patellar, Right	10 (63)	12 (80)	.283
Patellar, Left	11 (69)	13 (87)	.233
Achilles, Right	3 (19)	6 (40)	.193
Achilles, Left	0 (0)	3 (20)	.060
Plantar fascia, Right	0 (0)	1 (7)	.294
Plantar fascia, Left	0 (0)	0 (0)	NA
Sonographic Evidence of Enthesopathy			
Supraspinatus, Right	0 (0)	1 (7)	.294
Supraspinatus, Left	0 (0)	0 (0)	NA
Patellar, Right	2 (13)	5 (33)	.166
Patellar, Left	5 (31)	3 (20)	.474
Achilles, Right	12 (75)	8 (53)	.208
Achilles, Left	12 (75)	7 (47)	.106
Plantar fascia, Right	0 (0)	0 (0)	NA
Plantar fascia, Left	1 (6)	0 (0)	.325

MSK, musculoskeletal; LEAF-Q, Low Energy Availability in Females Questionnaire; LESS, Landing Error Scoring System.

^†^*P* values represent exploratory uncorrected Pearson *χ*^2^ comparisons of marginal pre-season vs. post-season frequencies (NA indicates statistic could not be calculated).

Across lower extremity (LE) and upper extremity (UE) analyses, clustering was substantial, with conditional *R*^2^ values markedly exceeding marginal *R*^2^ (LE ≈ 0.74–0.89 vs. ≈0.03–0.05; UE composite: 0.91 vs. 0.12) and high ICCs (LE: 0.69–0.88; UE: 0.90), indicating that performance variance was dominated by between-athlete differences; component outcomes showed similar clustering patterns (LE ICC ≈ 0.52–0.75). Continuous tendon CSA measures in the anterior knee and posterior ankle regions showed no consistent main effects and no consistent Time × tendon CSA interactions across limb-specific composite or component performance measures. Similarly, upper extremity models showed no main effects for Time and no evidence that composite or component supraspinatus tendon thickness modified CKCUE Stability Test performance (Table 3; Figure 1).

**Table 3:** Mixed-effects models of continuous outcomes across the competitive season.

Variable	Time	Tendon Measure	Time × Measure
Composite			
Right Lower Extremity Performance vs.…			
Proximal patellar tendon CSA, Right	3.31 (.093)†	0.69 (.415)	2.67 (.127)
Achilles tendon CSA, Right	0.63 (.444)	0.62 (.437)	1.12 (.309)
Left Lower Extremity Performance vs.…			
Proximal patellar tendon CSA, Left	0.19 (.669)	0.66 (.428)	0.61 (.452)
Achilles tendon CSA, Left	0.00 (.989)	0.02 (.899)	0.08 (.788)
Closed Kinetic Chain Upper Extremity Stability Test vs.…			
Supraspinatus tendon thickness, Average	0.33 (.580)	0.78 (.385)	0.01 (.926)
Component			
Right single-limb vertical jump vs.…			
Proximal patellar tendon, Right	1.61 (.226)	0.83 (.372)	1.06 (.321)
Achilles tendon, Right	0.24 (.632)	0.01 (.974)	0.53 (.479)
Right single-limb hop vs.…			
Proximal patellar tendon, Right	1.33 (.270)	0.09 (.757)	1.09 (.316)
Achilles tendon, Right	0.35 (.562)	1.92 (.179)	0.64 (.437)
Left single-limb vertical jump vs.…			
Proximal patellar tendon, Left	0.01 (.910)	0.22 (.648)	0.45 (.517)
Achilles tendon, Left	0.00 (.986)	0.02 (.963)	0.15 (.711)
Left single-limb hop vs.…			
Proximal patellar tendon, Left	1.45 (.254)	1.08 (.316)	8.42 (**.014**)
Achilles tendon, Left	0.01 (.915)	0.46 (.504)	0.65 (.435)
Closed Kinetic Chain Upper Extremity Stability Test vs.…			
Supraspinatus tendon thickness, Right	0.22 (.646)	1.11 (.301)	0.05 (.834)
Supraspinatus tendon thickness, Left	0.61 (.453)	0.16 (.693)	0.00 (.971)

CSA, cross-sectional area.

Values are F (*P* value).

Bold indicates *P*< .05.

† *P* < .10, exploratory trend.

**Figure 1:**
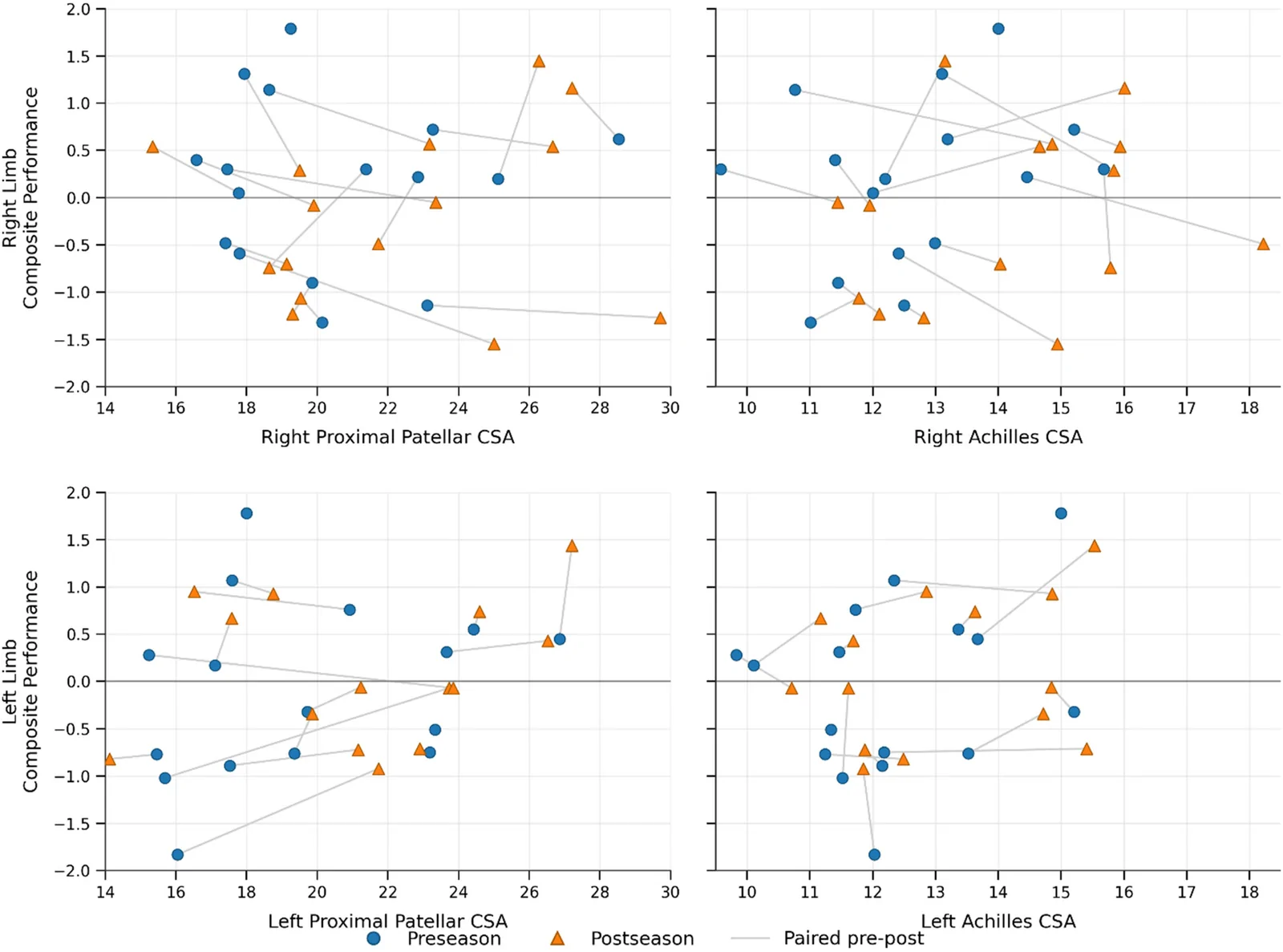
Limb-specific relationships between sonographic tendon structure and composite performance measures across a competitive volleyball season. Composite performance scores are standardized (mean = 0). Tendon cross-sectional area (CSA) values are normalized.

Categorical pathology models (i.e., tendinopathy and/or enthesopathy) showed no consistent main effects and no Time × pathology interactions; only the left limb patellar pathology model showed a significant Time effect (estimate = 0.35, 95% CI 0.06–0.64; *P* = .022), indicating an overall pre-season vs. post-season change regardless of pathology status. Neither the main effect of pathology nor the Time × pathology interaction was significant (*P* > .260). Right limb patellar pathology showed trend level Time (F = 3.85; *P* = .070). Overall, conditional *R*^2^ values were high (≈0.85–0.90) and marginal *R*^2^ values remained low (<0.06), consistent with strong athlete-level clustering and limited fixed effect contribution.

Within-session reliability was excellent for most performance measures (ICC_3,3_ = 0.94–0.98), good for the CKCUE stability test (ICC_3,2_ = 0.86), and moderate-to-good for left single-leg hop (ICC_3,3_ = 0.69). Complete reliability estimates are provided in Table 4.

**Table 4:** Within-session reliability of pre-season performance measures.

Measure	Trials (k)	N	ICC_3,k_	95% CI
Right single-limb hop	3	31	0.96	0.93, 0.98
Left single-limb hop	3	30	0.69	0.42, 0.84
Right single-limb vertical jump	3	31	0.95	0.91, 0.97
Left single-limb vertical jump	3	30	0.95	0.90, 0.97
Broad jump	3	31	0.95	0.90, 0.97
Double broad jump	3	30	0.94	0.89, 0.97
Vertical jump	3	31	0.98	0.95, 0.99
Drop jump	3	31	0.96	0.93, 0.98
CKCUE stability test	2	31	0.86	0.63, 0.94

ICC, intraclass correlation coefficient; CI, confidence interval; CKCUE, closed kinetic chain upper extremity.

### Secondary analysis

Secondary models tested sonographic asymmetry and contextual predictors (LESS score, RESTQ-36-Sport, LEAF-Q) for double-limb composite and component performance measures. Across all double-limb jump-based outcomes, ICC values remained moderate to high (≈0.62–0.82), and marginal *R*^2^ values were low (<0.10), indicating that fixed effects contributed minimally to explaining performance variability relative to athlete-level effects.

### Correlations

Nonparametric correlations were used to examine associations between sonographic tissue structure and performance measures across the season. Correlation coefficients were generally small to moderate in magnitude (|*ρ*| < 0.45) and did not reach statistical significance (all *P* > .110), indicating weak or absent monotonic relationships between tissue adaptation and performance change. Changes in left and right Achilles tendon CSA demonstrated large magnitude correlations (*ρ* approximately −0.69 to 0.75; *P* ≤ 0.01), indicating that athletes who exhibited greater structural change on one side tended to show concordant changes on the contralateral side.

## Discussion

Across a competitive season for athletes on a single Division I female volleyball team, several key findings emerged. First, performance measures demonstrate modest and variable pre-season vs. post-season changes, consistent with task-specific adaptation rather than uniform performance decline or improvement. Second, continuous and categorical sonographic tissue characteristics were not meaningfully associated with either the primary composite performance outcomes or the individual component performance measures, indicating that sonographic tissue characteristics did not explain inter-individual differences in performance or its seasonal trajectory. This pattern is consistent with prior observations in elite jumping athletes, where structural tendon adaptations or pathology have been shown to coexist with preserved or enhanced jump performance, suggesting that tissue morphology and functional capacity may not be tightly coupled in trained populations (32, 33). Third, contextual monitoring variables (LESS score and athlete-reported RESTQ-36-Sport and LEAF-Q scores) were not associated with double-limb performance measures and did not moderate seasonal changes in key performance variables. The present findings are particularly relevant to female volleyball athletes, a population in which athlete monitoring strategies frequently incorporate measures related to connective tissue health, movement quality, and energy availability. Despite the unique physiological and sport-specific demands experienced by female athletes, sonographic tissue characteristics remained weakly associated with functional performance outcomes. These findings suggest that structural imaging findings should be interpreted within a broader athlete-monitoring framework rather than as isolated indicators of performance capacity in female volleyball athletes.

Because the limb-specific composite performance scores were designated *a priori* as the primary outcome measure, these findings warrant consideration. The composite scores were intended to provide a broader representation of lower extremity explosive performance by integrating performance across both single-leg hop distance and single-leg vertical jump height while reducing the number of primary statistical comparisons. Across all models, neither continuous tendon morphology measures nor categorical pathology indicators demonstrated meaningful associations with composite performance scores or composite performance trajectories across the season. The consistently low marginal *R*^2^ values and absence of significant Time × tissue interactions suggest that sonographic tissue characteristics explained little of the variability in overall lower extremity performance adaptation. These findings support the interpretation that season long changes in composite performance were primarily driven by athlete-specific factors rather than measured sonographic tissue characteristics.

Comparison with prior literature should also account for differences in sex and sport exposure across study populations. Previous investigations have included mixed sport collegiate cohorts, dancers, distance runners, and elite jumping athletes, and some reports have not provided sex-specific estimates (2, 9, 24, 25, 32, 33). Therefore, differences in loading exposure, competitive level, and participant sex may limit direct comparison with the present single team cohort of Division I female volleyball athletes. The current findings extend this literature by evaluating sonographic structure and performance concurrently and longitudinally in a specifically defined female athlete population.

An exploratory association was observed between LESS scores and vertical jump height, suggesting a potential cross sectional relationship between jump-landing movement quality and performance capacity; however, the absence of significant interaction effects indicates that LESS scores did not influence the direction or magnitude of performance change across the season (20). Collectively, these findings support a lack of coupling between sonographic structural adaptation and functional performance change (8, 10). Importantly, much of the USI literature has focused on predicting future pain or injury rather than explaining contemporaneous performance, and meta-analytic evidence indicates that while sonographic abnormalities may increase future pain risk, their positive predictive value is low and does not imply current functional impairment (9, 11, 13).

Consistent with prior work, these findings support interpreting sonographic abnormalities within the broader clinical and performance context rather than using USI examination findings in isolation (5, 8). Exploratory correlation analyses further demonstrated that, while sonographic tissue characteristics exhibited strong bilateral coupling, particularly in the Achilles tendon, these structural changes were weakly related to performance adaptations. Such bilateral coupling aligns with sport-specific tendon adaptation profiles described in highly trained athletes, where tendon size and morphology appear to adjust to cumulative loading demands rather than limb-specific functional output (25). Taken together, the results suggest that tissue structural changes and performance adaptations may follow partially independent trajectories over the course of a single competitive season.

This study should be interpreted considering several limitations. First, this pilot investigation included a relatively small sample of Division I female volleyball athletes from a single team, which limited statistical power to evaluate interaction effects, restricts generalizability beyond similar athletic populations. Second, the approach used to describe and categorize sonographic bony abnormalities was exploratory and was not intended to establish clinical diagnostic thresholds; the clinical relevance of these findings remains uncertain, particularly given heterogeneity in sonographic-based definitions and grading criteria across studies (15, 29). The present study was also limited to conventional grayscale measures of tendon morphology and qualitative pathology and did not quantify echointensity or tendon mechanical properties using shear wave elastography. These techniques may provide complementary information regarding tissue composition and stiffness, although shear wave elastography measurements are sensitive to positioning, loading, acquisition protocol, and equipment their relationships with sport-specific functional performance require further longitudinal investigation (34, 35). The imaging protocol was limited to selected tendons, entheses, and the plantar fascia and did not include sonographic measures of muscle morphology or quality, such as muscle thickness, CSA, pennation angle, or echointensity. Because muscle architecture contributes to force and power production, future studies should evaluate muscle and tendon characteristics concurrently when examining relationships with functional performance (36). Third, the absence of a control group (e.g., non-jumping or non-hitting athletes) precludes direct comparison of seasonal sonographic tissue changes across athletic exposures, limiting inferences regarding sport-specific adaptation. Additionally, sonographic and performance measurements were collected only at pre-season and post-season time points. Consequently, transient changes occurring during the competitive season may not have been captured, limiting insight into the temporal trajectory of tissue adaptation and performance fluctuations throughout the season. Fourth, study-specific intra-rater reliability was not established for the sonographic measurements, although procedural variability was reduced through standardized acquisition and analysis by a single experienced examiner. Despite these limitations, the repeated measures design, standardized imaging protocols, and use of mixed-effects modeling strengthen internal validity and support the robustness of the observed null and exploratory findings.

## Conclusion

In Division I female volleyball athletes, seasonal performance adaptations were modest and task-specific, while sonographic tissue characteristics were not meaningfully associated with performance level or longitudinal performance change. Despite strong bilateral coupling of tendon structure, particularly within the Achilles tendon, these structural characteristics showed weak correspondence with functional performance adaptations, suggesting that tissue structural adaptation and performance adaptation may occur along partially independent pathways over a competitive season. From a sport science and athlete monitoring perspective, these findings caution against interpreting isolated sonographic abnormalities as indicators of reduced performance or maladaptation in asymptomatic athletes. Instead, sonographically derived tissue characteristics should be interpreted within a broader contextual framework that includes performance testing, movement quality, training exposure, and athlete reported factors.

## Data Availability

The raw data supporting the conclusions of this article will be made available by the authors, without undue reservation.
